# Supplementary material for: Isolation and Characterization of a Novel Pathogenesis-Related Protein-1 Gene (AvPR-1) with Induced Expression in Oat (Avena sativa L.) during Abiotic and Hormonal Stresses
Source: Plants (Basel). 2022 Aug 31;11(17):2284. doi: 10.3390/plants11172284 (PMC9460936; doi:10.3390/plants11172284)
Supplement: Supplementary file 1 [file plants-11-02284-s001.zip › plants-1878820-supplementary.pdf]

Supplemental Figure S1

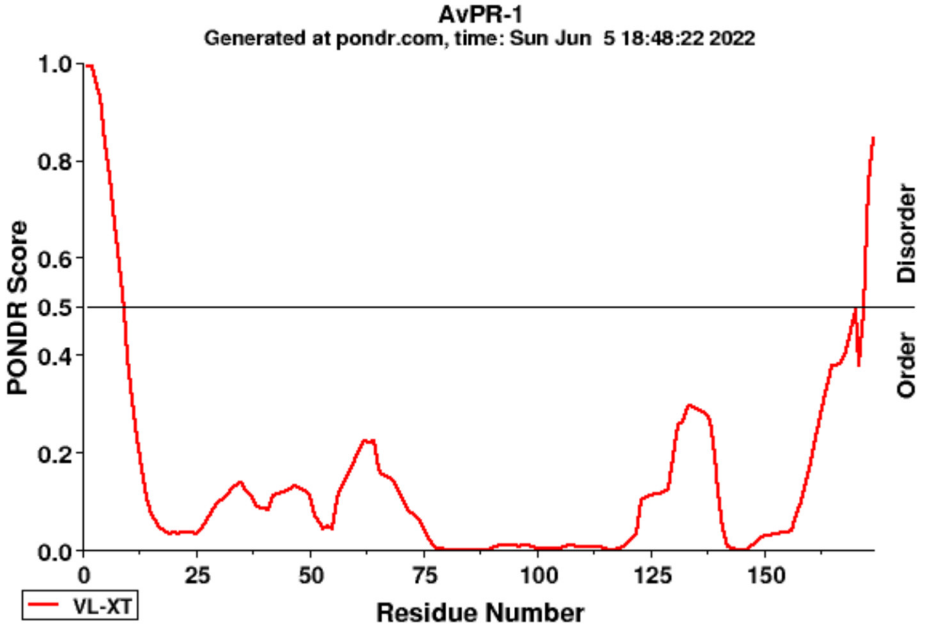

Supplemental Figure S1: Identification of signal peptides in AvPR 1 sequence using PONDR (Predictor of Natural Disordered Regions) server (http:://www pondr.com/) database.

Supplemental Figure S2

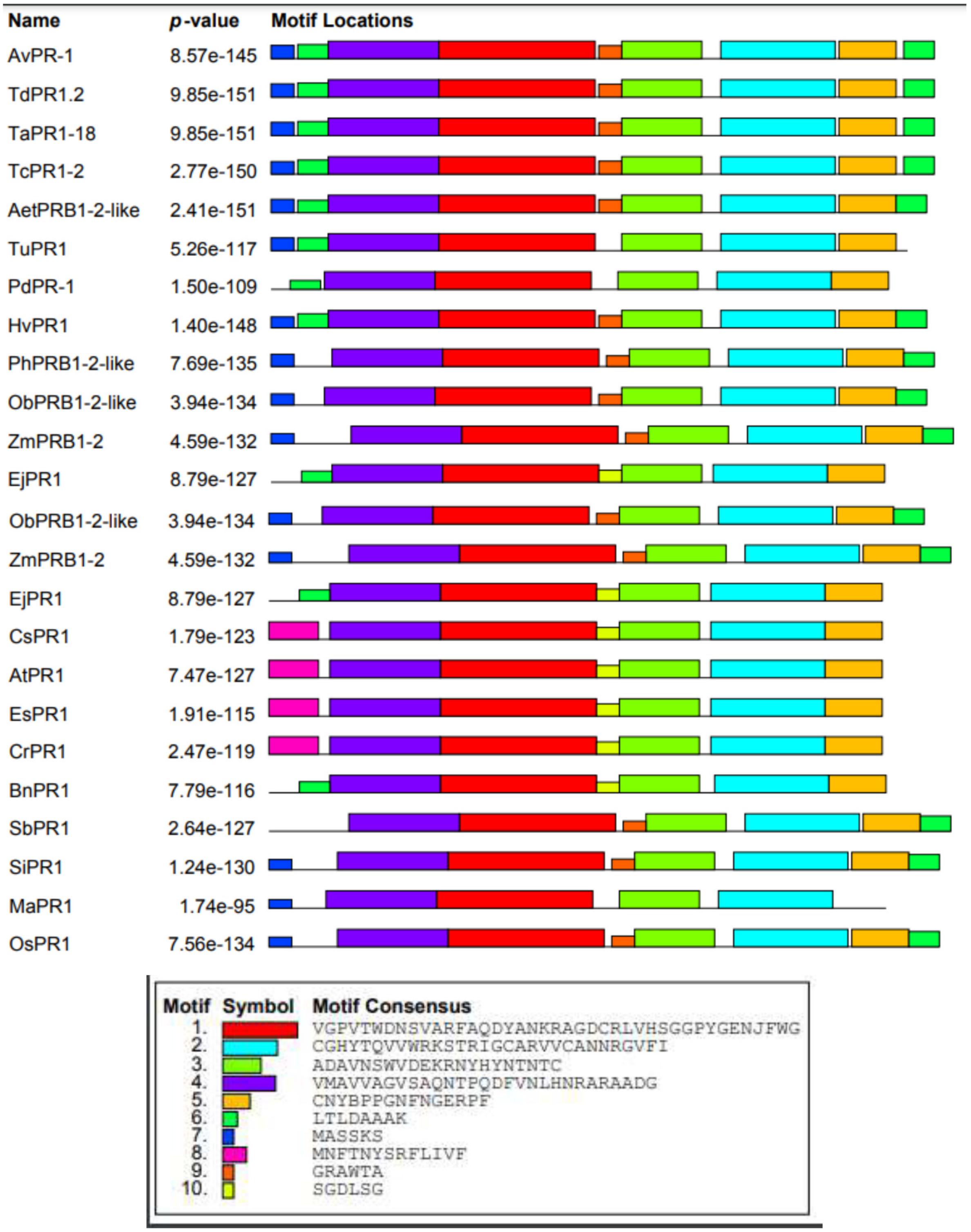

Supplemental Figure S2: Conserved motifs identified from AvPR-1 protein and its homologsin plants.

Supplemental Figure S3

```
#
    MASSKSSLAMFALAIVMAVVAGRSAQNTPQDFVNLHNRARAGDGVGPVTW   #     50
    DNSVARFAQDYANKRAADCRLQHSGAPFGENIFWGSGQSWTAANAVTSWV   #    100
    DEKRNYHLNTNTCDAGKVCGHYTQVVWRKSTRIGCARVVCAGNRGVFITC   #    150
    NYNPPGNFNGERPFAFLTLDAEAK                             #    200
%1  ..SS..S................S...T....................T.   #     50
%1  ..........Y........................S..S........S..   #    100
%1  .............................ST...................   #    150
%1  ........................
```

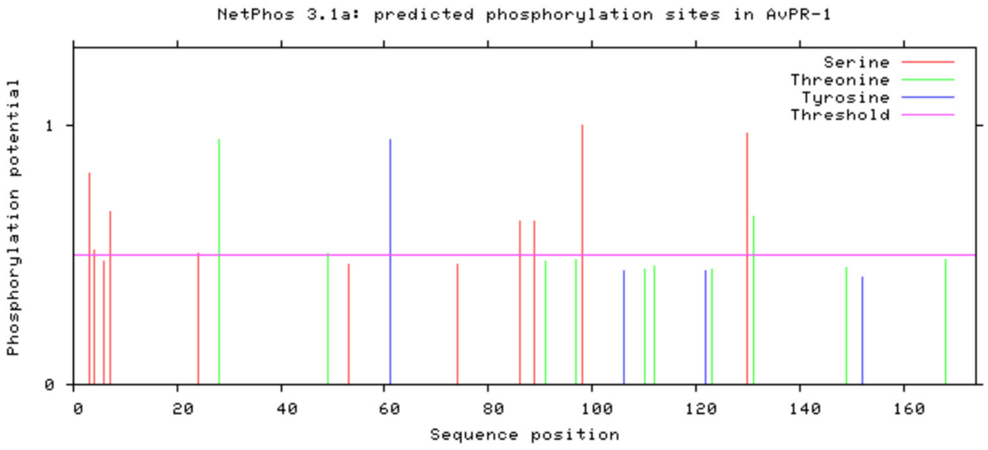

Supplemental Figure S3: Identification of putative phosphorylation sites as revealed by NetPhos database.

Supplemental Figure S4

Query header	gene name	Description Estimated PPV, description		Biological process Estimated PPV, GO-id, description			Molecular function Estimated PPV, GO-id, description	Cellular component Estimated PPV, GO-id, description		
AvPR-1 AvPR-1 Search		0.87	Pathogenesis-related protein 1-5	0.66	GO:0009607	response to biotic stimulus		0.65	GO:0005576	extracellular region
				0.37	GO:0006952	defense response		0.32	GO:0016021	integral component of membrane

Supplemental Figure S4: Identification of the biological role of AvPR-1 as revealed bythePANNZER2 online server.
